# Association between remnant cholesterol and arterial stiffness in a Chinese community-based population: A cross-sectional study

**DOI:** 10.3389/fcvm.2022.993097

**Published:** 2022-11-11

**Authors:** Jiahui Liu, Fangfang Fan, Bo Liu, Kaiyin Li, Yimeng Jiang, Jia Jia, Chuyun Chen, Bo Zheng, Yan Zhang

**Affiliations:** Department of Cardiology, Institute of Cardiovascular Disease, Peking University First Hospital, Beijing, China

**Keywords:** brachial-ankle pulse wave velocity, arterial stiffness, remnant cholesterol, lipids, cross-sectional study

## Abstract

**Objectives:**

As a surrogate of arterial stiffness, the brachial-ankle pulse wave velocity (baPWV) is a good predictor of incident cardiovascular disease. Remnant cholesterol (RC) is a proven independent risk factor for cardiovascular disease. However, the relationship between RC and baPWV is unknown. The present study was performed to explore this relationship.

**Design:**

Cross-sectional study.

**Setting and participants:**

This study involved 8,028 participants of a community-based atherosclerosis cohort from China. Community residents aged ≥40 years were enrolled by responding to detailed research recruitment posters or by phone invitation. The participants comprised 2,938 (36.60%) men, and their mean age was 56.57 ± 9.04 years.

**Methods and results:**

The baPWV was measured with a standard protocol using the Omron Colin BP-203RPE III device (Omron Healthcare, Kyoto, Japan). RC was calculated as follows: RC = TC – LDL-C – HDL-C. The mean baPWV was 1,646.85 ± 374.11 cm/s. The median RC concentration was 0.56 (0.41–0.74) mmol/L. In the multivariate logistic regression analyses, the concentrations of RC, triglycerides (TG), total cholesterol (TC), low-density lipoprotein cholesterol (LDL-C), and non-high-density lipoprotein cholesterol (non-HDL-C) were all positively and independently associated with baPWV. The baPWV was higher in the fourth than first lipid profile quartile. The HDL-C concentration was inversely associated with baPWV. When RC was forced into the model with other lipid profile indices simultaneously, only the RC and TG concentrations remained significantly associated with baPWV.

**Conclusion:**

Lipids are independently associated with baPWV. The RC and TG concentrations have stronger associations with arterial stiffness than other lipid indices in the Chinese community-based population.

## Introduction

Arterial stiffness is a well-recognized independent risk factor for the development of cardiovascular disease (CVD) (1). Increased arterial stiffness intensifies the pulsatile blood flow and strains the arterial wall, which contributes to the development of atherosclerosis (2). Many studies have proved that increased arterial stiffness predicts all-cause and cardiovascular mortality (1, 3–5). As a convenient and noninvasive measurement index, the brachial-ankle pulse wave velocity (baPWV) is widely used for precise assessment of arterial stiffness (5). Increased age and blood pressure are considered major factors associated with baPWV (4). However, the relationship between baPWV and other important modifiable risk factors for CVD, such as dyslipidemia, is ambiguous.

Remnant cholesterol (RC) is the cholesterol component of triglyceride-rich lipoproteins, which are composed of very-low-density lipoproteins and intermediate-density lipoproteins in the fasting state and both of these lipoproteins together with chylomicron remnants in the non-fasting state (6). Previous researches have suggested that RC is an important contributor to atherosclerosis and development of CVD (6–8). One study showed that individuals at high risk of CVD had a 21% higher risk of major adverse cardiac events per 10-mg/dL increase in RC (9).

A few studies have focused on the association between the lipids and baPWV, but the results are controversial (10–12). Therefore, we investigated the associations of RC and other lipid indices with baPWV and determined which association is strongest among community dwellers in the general Chinese population.

## Materials and methods

### Study population

The data used in this cross-sectional study originated from an atherosclerosis cohort that was analyzed from December 2011 to April 2012 in the Gucheng and Pingguoyuan communities of Shijingshan District in Beijing, China (13). Community residents aged ≥40 years were enrolled by responding to detailed research recruitment posters or by phone invitation. Written informed consent was obtained from all participants, and the study was approved by the Institutional Review Board of Peking University First Hospital. We adhered to the principles of the Declaration of Helsinki. The procedures followed were in accordance with institutional guidelines.

### Data collection

We conducted a standardized questionnaire to collect information including age, sex, lifestyle, cardiovascular risk factors, and medical history. Trained physicians obtained the participants' anthropometric measurements such as height, weight, waist circumference, hip circumference, and blood pressure using standard methods.

After an overnight fast of at least 12 h, a venous blood sample was obtained from the forearm of each participant. The fasting serum total cholesterol (TC), triglyceride (TG), high-density lipoprotein cholesterol (HDL-C), low-density lipoprotein cholesterol (LDL-C), creatinine, alanine aminotransferase, aspartate aminotransferase, and fasting blood glucose concentrations were measured on a Roche cobas^®^ 8000 Automatic Analyzer (Roche Diagnostics, Basel, Switzerland). The TG, RC, HDL-C and LDL-C were measured directly. RC was calculated as follows: RC = TC – LDL-C – HDL-C (14). Non-HDL-C was calculated as follows: non-HDL-C = TC – HDL-C. The glomerular filtration rate (GFR) (expressed in mL/min/1.73 m^2^) was calculated by the Chronic Kidney Disease Epidemiology Collaboration equation.

A trained technician used a standard protocol to measure baPWV with the participants in the supine position after a 5-min rest using the Omron Colin BP-203RPE III device (Omron Healthcare, Kyoto, Japan). The bilateral baPWVs were automatically calculated by the instrument, and the higher of the two was recorded for subsequent analyses.

### Statistical analysis

Current smoking and drinking were defined as smoking one cigarette per day and drinking once per week, respectively, for at least 6 months. The body mass index (BMI) was calculated as the weight in kilograms divided by the square of the height in meters. Hypertension was defined as systolic blood pressure of ≥140 mmHg, diastolic blood pressure of ≥90 mmHg, or self-reported history of hypertension or taking antihypertensive agents. Diabetes mellitus was defined as a fasting blood glucose concentration of ≥7.0 mmol/L or self-reported history of diabetes mellitus, taking antiglycemic agents, or undergoing insulin therapy. Lipid-lowering drugs in this study referred to both TG-lowering drugs and cholesterol-lowering drugs.

Continuous variables are expressed as mean ± standard deviation or median (interquartile range). Categorical variables are presented as frequency and percentage. Normally distributed variables were compared using the *t*-test, and non-normally distributed variables were compared using the Kruskal-Wallis test. Categorical variables were compared using the Pearson chi-square test or Fisher's exact test. Multivariable logistic regression models were used to investigate the association between baPWV and lipid profiles after adjusting for age, sex, current smoking and drinking habits, BMI, hypertension, and diabetes mellitus. A two-tailed *P* < 0.05 was considered statistically significant in all analyses. R software (version 3.4.3, http://www.R-project.org) and Empower (R; www.empowerstats.com, X&Y Solutions, Inc., Boston, MA, USA) were used for all statistical analyses.

## Results

### Baseline characteristics of study participants

This cross-sectional study initially involved 9,540 participants. Participants with missing baPWV data (*n* = 385), with missing lipid profiles (*n* = 37), and who were taking lipid-lowering medications (*n* = 1,090) were excluded. Thus, 8,028 community-dwellers were included in the final analysis. Among them, 2,938 (36.60%) were male and 5,090 (63.40%) were female. Their mean age was 56.57 ± 9.04 years. A total of 3,693 (46.00%) participants had hypertension, 1,864 (23.22%) were diagnosed with diabetes, and only 70 (0.87%) had a history of myocardial infarction. Of all surveyed community-dwellers, 1,574 (19.61%) were current smokers and 1,921 (23.94%) were current alcohol drinkers. In total, 197 (2.46%) participants were diagnosed with peripheral artery disease, which was based on an ankle-brachial index of ≤0.90 in our study. A total of 272 (2.39%) participants had a self-reported stroke history. The mean baPWV was 1,646.85 ± 374.11 cm/s. The median RC and TG concentrations were 0.56 (0.41–0.74) mmol/L and 1.28 (0.91–1.83) mmol/L, respectively, and the mean LDL-C concentration was 3.28 ± 0.82 mmol/L. We further assigned the participants into subgroups using RC quartiles. In the highest RC group, the baPWV, TC, TG, LDL-C, and non-HDL-C were significantly higher than those in the lowest RC group (*P* < 0.05). Similarly, the proportions of hypertension, diabetes, myocardial infarction history, smoking, and drinking were higher in the highest RC group than in the lowest RC group. In contrast, the estimated GFR, HDL-C concentration, and proportion of patients with a stroke history decreased from the highest RC group to the lowest RC group (Table 1).

**Table 1:** Characteristics of the study participants.

**Variables**	**Total**	**RC Quartiles, mmol/L**				***P*-value**
		**(< 0.41)**	**(0.41–0.56)**	**(0.56–0.74)**	**(≥0.74)**	
*N*	8,028	2,006	2,005	1,988	2,029	
Male, *n* (%)	2,938 (36.60)	638 (31.80)	726 (36.21)	714 (35.92)	860 (42.39)	<0.001
Age, years	56.57 ± 9.04	55.81 ± 9.34	56.87 ± 9.51	57.17 ± 8.73	56.44 ± 8.49	<0.001
BMI, kg/m^2^	25.93 ± 3.42	24.49 ± 3.18	25.74 ± 3.39	26.52 ± 3.42	26.99 ± 3.16	<0.001
baPWV, cm/s	1646.85 ± 374.11	1566.06 ± 373.14	1629.54 ± 355.96	1679.02 ± 380.17	1712.30 ± 370.73	<0.001
RC, mmol/L	0.56 (0.41–0.74)	0.32 (0.25–0.36)	0.48 (0.44–0.52)	0.63 (0.59–0.68)	0.91 (0.81–1.10)	<0.001
TC, mmol/L	5.35 ± 0.98	4.75 ± 0.76	5.14 ± 0.82	5.52 ± 0.84	5.97 ± 1.04	<0.001
TG, mmol/L	1.28 (0.91–1.83)	0.80 (0.65–0.99)	1.13 (0.91–1.34)	1.49 (1.20–1.80)	2.36 (1.82–3.09)	<0.001
HDL-C, mmol/L	1.44 ± 0.38	1.71 ± 0.38	1.49 ± 0.34	1.37 ± 0.31	1.20 ± 0.28	<0.001
LDL-C, mmol/L	3.28 ± 0.82	2.74 ± 0.61	3.17 ± 0.65	3.51 ± 0.69	3.70 ± 0.95	<0.001
Non-HDL-C, mmol/L	3.90 ± 0.98	3.04 ± 0.64	3.65 ± 0.66	4.15 ± 0.70	4.77 ± 0.94	<0.001
eGFR, ml/min per 1.73 m^2^	94.96 ± 13.01	96.94 ± 12.21	94.90 ± 13.08	94.11 ± 13.11	93.90 ± 13.38	<0.001
Smoking, *n* (%)	1,574 (19.61)	264 (13.16)	350 (17.46)	385 (19.37)	575 (28.34)	<0.001
Drinking, *n* (%)	1,921 (23.94)	414 (20.65)	463 (23.09)	478 (24.06)	566 (27.91)	<0.001
Hypertension, *n* (%)	3,693 (46.00)	741 (36.94)	899 (44.84)	961 (48.34)	1,092 (53.82)	<0.001
Diabetes, *n* (%)	1,864 (23.22)	335 (16.70)	401 (20.00)	486 (24.45)	642 (31.64)	<0.001
Self-reported MI, *n* (%)	70 (0.87)	8 (0.40)	15 (0.75)	23 (1.16)	24 (1.18)	<0.001
Self-reported Stroke, n (%)	272 (3.39)	58 (2.89)	87 (4.34)	69 (3.47)	58 (2.86)	<0.001

Continuous variables are presented as median (interquartile range) or mean ± standard deviation, and dichotomous variables are presented as frequency (percentage).

Q, quartile; BMI, body mass index; eGFR, estimated glomerular filtration rate; MI, myocardial infarction; baPWV, brachial-ankle pulse wave velocity; TC, total cholesterol; TG, triglyceride; HDL-C, high-density lipoprotein cholesterol; LDL-C, low-density lipoprotein cholesterol; RC, remnant cholesterol.

### Associations between lipid profiles and baPWV

**Figure 1:**
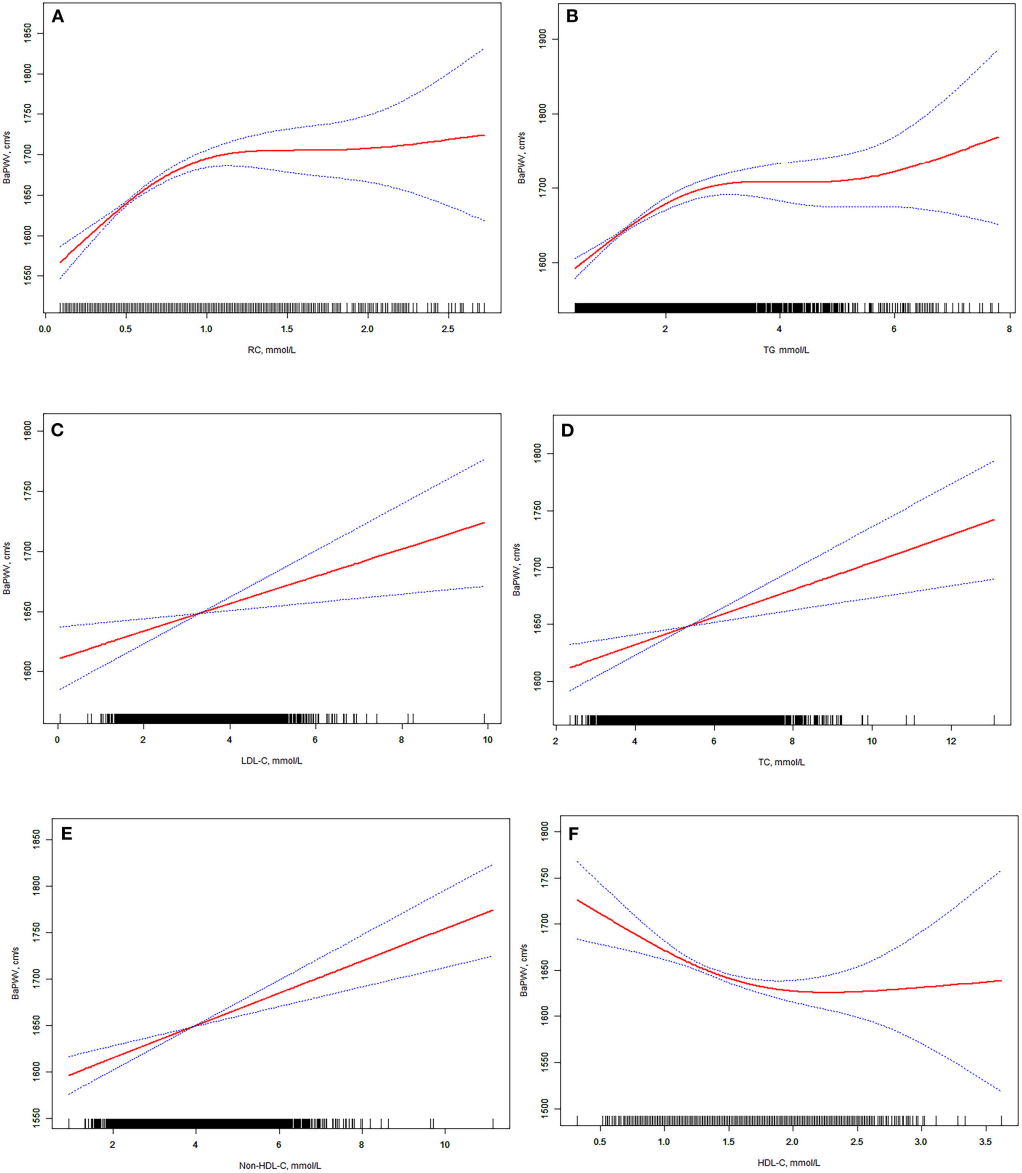
**(A–F)** Associations between brachial-ankle pulse wave velocity and lipid profiles. We conducted restricted cubic spline curves to evaluate non-linear relationship between lipids and baPWV. After adjustment for covariables including sex, age, BMI, eGFR, smoking, drinking, hypertension history, diabetes history, myocardial infarction history, and stroke history, the significant associations between lipid profiles and baPWV remained statistically significant. 95% confidence interval is shown in blue. BMI, body mass index; eGFR, estimated glomerular filtration rate; CI, confidence interval; baPWV, brachial-ankle pulse wave velocity; TC, total cholesterol; TG, triglyceride; HDL-C, high-density lipoprotein cholesterol; LDL-C, low-density lipoprotein cholesterol; RC, remnant cholesterol.

We applied a smoothing plot to examine the relationships between lipid profiles and baPWV. After adjusting for potential confounders (including sex, age, BMI, estimated GFR, smoking, drinking, hypertension, diabetes, stroke, and myocardial infarction), non-linear relationships between lipid profiles and baPWV were observed (Figure 1).

As shown in Table 2, the RC, TG, TC, LDL-C, and non-HDL-C concentrations were all independently and positively associated with baPWV after adjustment for various confounders, and the HDL-C concentration was negatively associated with baPWV (all *P* < 0.05). Participants were stratified by quartiles of RC concentrations, and when we used the lowest quartile (Q1) as a reference, the β value increased along with the RC concentration [second quartile (Q2): β = 25.41, 95% confidence interval (CI): 7.43–43.38, *P* = 0.006; third quartile (Q3): β = 59.58, 95% CI: 41.25–77.91, *P* < 0.001; highest quartile (Q4): β = 86.34, 95% CI: 67.63–105.06, *P* < 0.001]. Meanwhile, participants with higher TG, LDL-C, TC, and non-HDL-C concentrations had higher baPWV, and a stronger relationship between lipid profiles and baPWV was found in the higher quartile groups than in the lowest quartile (all *P* < 0.05). In addition, the HDL-C concentration was negatively associated with baPWV. Furthermore, after adjustment for covariables including sex, age, BMI, GFR, smoking and drinking habits, hypertension, diabetes mellitus, and myocardial infarction and stroke history, the significant association between lipid profiles and baPWV was still statistically significant (Table 2). The subgroup analysis results were in accordance with the main analysis results.

**Table 2:** Associations between brachial-ankle pulse wave velocity and each lipid index.

**Lipids**	**Crude model**		**Multivariable adjusted model** *	
	**β (95% CI)**	***P*-value**	**β (95% CI)**	***P*-value**
RC, mmol/L				
Q1 (< 0.41)	0		0	
Q2 (0.41–0.56)	63.48 (40.58, 86.39)	<0.001	25.41 (7.43, 43.38)	0.006
Q3 (0.57–0.74)	112.96 (90.00, 135.91)	<0.001	59.58 (41.25, 77.91)	<0.001
Q4 (≥0.74)	146.24 (123.40, 169.08)	<0.001	86.34 (67.63, 105.06)	<0.001
P for trend		<0.001		<0.001
LDL-C Quartiles, mmol/L				
Q1 ( ≤ 2.72)	0		0	
Q2 (2.73–3.23)	12.33 (-10.77, 35.44)	0.295	6.34 (-11.58, 24.26)	0.488
Q3 (3.24–3.78)	50.12 (26.96, 73.28)	<0.001	19.93 (1.89, 37.98)	0.030
Q4 (≥3.79)	71.12 (47.96, 94.28)	<0.001	24.36 (6.10, 42.63)	0.009
*P* for trend		<0.001		0.003
TG Quartiles, mmol/L				
Q1 ( ≤ 0.90)	0		0	
Q2 (0.91–1.27)	82.58 (59.64, 105.52)	<0.001	28.86 (10.84, 46.88)	0.002
Q3 (1.28–1.82)	119.40 (96.43, 142.37)	<0.001	49.93 (31.57, 68.29)	<0.001
Q4 (≥1.83)	153.88 (130.90, 176.86)	<0.001	88.99 (70.16, 107.82)	<0.001
*P* for trend		<0.001		<0.001
HDL-C Quartiles, mmol/L				
Q1 ( ≤ 1.16)	0		0	
Q2 (1.17–1.39)	−16.26 (−39.33, 6.81)	0.167	−10.45 (−28.67, 7.78)	0.261
Q3 (1.40–1.65)	−72.05 (−95.20, −48.91)	<0.001	−38.46 (−57.30, −19.62)	<0.001
Q4 (≥1.66)	−99.58 (−122.73, −76.42)	<0.001	−45.32 (−65.23, −25.42)	<0.001
*P* for trend		<0.001		<0.001
TC Quartiles, mmol/L				
Q1 ( ≤ 4.69)	0		0	
Q2 (4.70–5.27)	−10.34 (−33.47, 12.80)	0.381	4.21 (−13.75, 22.16)	0.646
Q3 (5.28–5.94)	34.06 (11.00, 57.11)	0.004	27.15 (9.14, 45.16)	0.003
Q4 (≥5.95)	59.86 (36.78, 82.94)	<0.001	30.40 (12.13, 48.67)	0.001
*P* for trend		<0.001		<0.001
Non-HDL-C Quartiles, mmol/L				
Q1 ( ≤ 3.23)	0		0	
Q2 (3.23–3.84)	37.16 (14.11, 60.20)	0.002	17.92 (0.00, 35.83)	0.050
Q3 (3.85–4.50)	61.73 (38.67, 84.80) 1	<0.001	24.69 (6.63, 42.75)	0.007
Q4 (≥4.51)	102.20 (79.18, 125.22)	<0.001	48.27 (30.05, 66.49)	<0.001
*P* for trend		<0.001		<0.001

* Adjusted for sex, age, BMI, eGFR, smoking, drinking, hypertension and antihypertensive drugs, diabetes and hypoglycemic drugs, stroke history, and myocardial infarction history.

CI, confidence interval; Q, quartile; BMI, body mass index; eGFR, estimated glomerular filtration rate; TC, total cholesterol; TG, triglyceride; HDL-C, high-density lipoprotein cholesterol; LDL-C, low-density lipoprotein cholesterol; RC, remnant cholesterol.

When RC was forced into the multivariable logistic regression model that was used to investigate the association between baPWV and lipid profiles with other lipid profile indices simultaneously, only the RC and TG concentrations were still significantly associated with baPWV after adjusted for sex, age, BMI, eGFR, smoking, drinking, hypertension history, diabetes history, and myocardial infarction history. An association was not observed between baPWV and other lipid parameters, including the LDL-C, TC, and non-HDL-C concentrations (Table 3).

**Table 3:** Associations between brachial-ankle pulse wave velocity and lipids with RC and other lipids entered into the model simultaneously.

**Variables**	**β (95% CI)**	***P*-value**	**Variables**	**β (95% CI)**	***P*-value**
**Comparison I** ^*^ **(when considered RC and LDL-C simultaneously)**					
RC, mmol/L			LDL-C, mmol/L
Q1 (<0.41)	0		Q1 ( ≤ 2.72)	0	
Q2 (0.41–0.56)	27.68 (9.14, 46.22)	0.003	Q2 (2.73–3.23)	−5.25 (−23.41, 12.91)	0.571
Q3 (0.56–0.73)	64.01 (44.26, 83.76)	<0.001	Q3 (3.24–3.78)	−2.72 (−21.60, 16.15)	0.777
Q4 (≥0.74)	92.14 (71.63, 112.66)	<0.001	Q4 (≥3.79)	−14.86 (−34.94, 5.21)	0.147
**Comparison II** ^*^ **(when considered RC and TG simultaneously)**					
RC, mmol/L			TG, mmol/L	
Q1 (< 0.41)	0		Q1 ( ≤ 0.90)	0	
Q2 (0.41–0.56)	16.05 (−3.39, 35.48)	0.106	Q2 (0.91–1.27)	18.70 (−0.45, 37.85)	0.056
Q3 (0.56–0.73)	39.99 (17.67, 62.31)	<0.001	Q3 (1.28–1.82)	25.51 (3.31, 47.71)	0.024
Q4 (≥0.74)	51.02 (23.87, 78.17)	<0.001	Q4 (≥1.83)	50.53 (23.34, 77.72)	<0.001
**Comparison III** ^*^ **(when considered RC and HDL-C simultaneously)**					
RC, mmol/L			HDL-C, mmol/L	
Q1 (< 0.41)	0		Q1 ( ≤ 1.16)	0	
Q2 (0.41–0.56)	23.69 (5.40, 41.98)	0.011	Q2 (1.17–1.39)	1.67 (−16.79, 20.12)	0.860
Q3 (0.56–0.73)	56.68 (37.41, 75.74)	<0.001	Q3 (1.40–1.65)	−13.84 (−13.56, 5.88)	0.169
Q4 (≥0.74)	81.38 (60.80, 101.95)	< 0.001	Q4 (≥1.66)	−8.16 (−29.88, 13.56)	0.462
**Comparison IV** ^*^ **(when considered RC and TC simultaneously)**					
RC, mmol/L			TC, mmol/L	
Q1 (<0.41)	0		Q1 ( ≤ 4.69)	0	
Q2 (0.41–0.56)	26.39 (8.05, 44.72)	0.005	Q2 (4.70–5.27)	−9.92 (−28.10, 8.25)	0.285
Q3 (0.56–0.73)	61.58 (42.13, 81.03)	<0.001	Q3 (5.28–5.94)	2.07 (−16.76, 20.91)	0.829
Q4 (≥0.74)	89.95 (69.05, 110.86)	<0.001	Q4 (≥5.95)	−11.20 (−31.55, 9.16)	0.281
**Comparison V** ^*^ **(when considered RC and Non-HDL-C simultaneously)**					
RC, mmol/L			Non-HDL-C, mmol/L
Q1 (< 0.41)	0		Q1 ( ≤ 3.23)	0	
Q2 (0.41–0.56)	29.43 (10.19, 48.67)	0.003	Q2 (3.23–3.84)	−2.52 (−21.46, 16.43)	0.795
Q3 (0.56–0.73)	66.93 (45.56, 88.30)	<0.001	Q3 (3.85–4.50)	−15.0 (−35.74, 5.71)	0.156
Q4 (≥0.74)	95.37 (71.60, 119.13)	<0.001	Q4 (≥4.51)	−11.42 (−34.49, 11.65)	0.332

* Adjusted for sex, age, BMI, eGFR, smoking, drinking, hypertension history, diabetes history, and myocardial infarction history.

CI, confidence interval; Q, quartile; BMI, body mass index; eGFR, estimated glomerular filtration rate; TC, total cholesterol; TG, triglyceride; HDL-C, high-density lipoprotein cholesterol; LDL-C, low-density lipoprotein cholesterol; RC, remnant cholesterol.

## Discussion

This study is the first to investigate the relationship between RC and baPWV in middle-aged and elderly community-dwellers in the Chinese general population. We found that the RC, TG, TC, LDL-C, and non-HDL-C concentrations were all independently and positively associated with arterial stiffness, whereas the HDL-C concentration showed an inverse association with arterial stiffness. Furthermore, RC and TG were stronger predictors of arterial stiffness than other lipid indices.

Previous studies have produced diverse results. Our findings are consistent with a study by Wang et al. (15) who performed a community-based cohort study in China with a mean follow-up of 4.8 years and found that a higher TG concentration was an independent predictor of baPWV. Additionally, Rider et al. (16) indicated that TG was related to increased arterial stiffness in adults with no identifiable cardiac risk factors. In contrast, a study of Chinese patients with hypertension showed that TG, TC, and non-HDL-C were positively related to baPWV, but the effect was not observed for LDL-C (11). In 429 healthy middle-aged women, no associations were observed between baPWV and HDL-C, TC, and TG (17). Wen et al. (18) found no significant association between high baPWV and TC, LDL-C, HDL-C, and non-HDL-C after adjusting for other variables in young Chinese men. These findings suggest that participants' disease history, race, sex, age, and sample size may affect the results.

No large study has been performed to evaluate the relationship between RC and arterial stiffness in Chinese dwellers. In the present study, RC and TG appeared to be superior to other traditional lipid parameters in assessing the risk of arterial stiffness. TG is independently related to insulin resistance, and insulin resistance leads to vascular stiffness in several ways. It plays a central role in activation of the renin–angiotensin–aldosterone system, reduction of bioavailable nitric oxide, and remodeling of the vascular extracellular matrix, all of which contribute to endothelial dysfunction, vessel wall hypertrophy and fibrosis, and vascular elasticity reduction (19).

Despite lowering LDL-C to the recommended level, patients with metabolic abnormalities still have considerable residual risks for cardiovascular events. Both clinical and genetic studies have proved that RC contributes to the residual risks (20, 21). RC is associated with increased risk of MI, ischemic heart disease, and death in the general population (22). Our study coincided with previous studies. Arterial stiffness is a subclinical CVD and highly associated with poor prognosis (23, 24). In our study, RC showed significantly predictive value for arterial stiffness, which may explain its residual risk for CVD. RC refers to the cholesterol content of a subset of the triglyceride-rich lipoproteins. Therefore, RC and TG are components of the same lipoproteins, their concentrations are highly correlated (25). The mechanism underlying how RC is related to arterial stiffness is unclear, but the above-mentioned studies involving TG are helpful. It is reported that TG lowering brings similar benefit to LDL-C levels per unit change in ApoB (26). Although TG showed positive association with arterial stiffness, RC as a part of atherogenic lipoprotein is more significant in CVD patients. The cholesterol content in RC can accumulate in atherosclerotic plaques. It can easily penetrate the vessel wall, cause inflammatory reactions (27), and activate the coagulation cascade (28). However, TG cannot directly lead to atherosclerotic plaques. TG-rich chylomicrons are too large to enter the arterial wall and become engulfed by vascular macrophages (29). Therefore, the estimation of RC, which is an easily obtained index in routine clinical practice and generally accepted in previous studies, may provide a reference for evaluating arterial stiffness.

### Limitations

This study has several limitations. First, it was an observational cross-sectional study. Although we performed a multivariable regression analysis by adjusting for several confounding factors, the current analysis was only hypothesis-generating; a further cohort study with follow-up data is needed to explore the causal link between RC and arterial stiffness. Second, the study only enrolled Chinese community-dwellers. Investigations in other populations are warranted to confirm the generalizability of our results. Third, our study relied on surrogate measures of RC instead of direct measurement. However, the indirect calculation of RC is generally accepted in previous studies (9, 30) and is recognized as an affordable and inexpensive method for clinical management. Finally, data on the use of antiplatelet medications were not collected, and the carotid intima-media thickness was measured in few participants. Thus, there might be confounding factors that were not included in our study.

## Conclusion

Lipid profiles are independently associated with baPWV, whereas RC and TG have stronger associations with arterial stiffness than do other lipid indices in the Chinese community-based population.

## Strengths and limitations of this study

This study is the first to investigate the relationship between RC and baPWV in middle-aged and elderly community-dwellers of the Chinese general population.

RC is an easily obtained index in routine clinical practice and may serve as a reference for evaluating arterial stiffness.

Although we performed a multiple regression analysis by adjusting for several confounding factors, the current analysis was only hypothesis-generating. A further cohort study with follow-up data is needed to explore the causal link between RC and arterial stiffness.

## Data availability statement

The raw data supporting the conclusions of this article will be made available by the authors, without undue reservation.

## Ethics statement

The studies involving human participants were reviewed and approved by Institutional Review Board of Peking University First Hospital. The patients/participants provided their written informed consent to participate in this study.

## Author contributions

BZ and YZ designed the study, revised the manuscript, and agree to be responsible for all aspects of the work. JL, JJ, YJ, CC, BL, and KL collected and re-checked the data. FF, JJ, and JL analyzed and interpreted the data. JL wrote the paper. All authors have read and approved the submitted manuscript.

## Funding

This study was supported by the UMHS-PUHSC Joint Institute for Translational and Clinical Research and the Fundamental Research Funds for the Central Universities (Grant No. BMU20160530), Capital's Funds for Health Improvement and Research (Grant No. 2020-2-2053), National Key Research and Development Program of China (Grant No. 2017YFC1307704), Projects of National Natural Science Foundation of China (Grant No. 81800312), Key Laboratory of Molecular Cardiovascular Sciences (Peking University) Ministry of Education, and NHC Key Laboratory of Cardiovascular Molecular Biology and Regulatory Peptides.

## Conflict of interest

The authors declare that the research was conducted in the absence of any commercial or financial relationships that could be construed as a potential conflict of interest.

## Publisher's note

All claims expressed in this article are solely those of the authors and do not necessarily represent those of their affiliated organizations, or those of the publisher, the editors and the reviewers. Any product that may be evaluated in this article, or claim that may be made by its manufacturer, is not guaranteed or endorsed by the publisher.
