# Supplementary material for: Association between remnant cholesterol and arterial stiffness in a Chinese community-based population: A cross-sectional study
Source: Front Cardiovasc Med. 2022 Nov 11;9:993097. doi: 10.3389/fcvm.2022.993097 (PMC9691684; doi:10.3389/fcvm.2022.993097)
Supplement: Supplementary file 1 [file Data_Sheet_1.docx]

Supplementary Figure 1. Subgroup and interaction analysis of association between RC and baPWV.


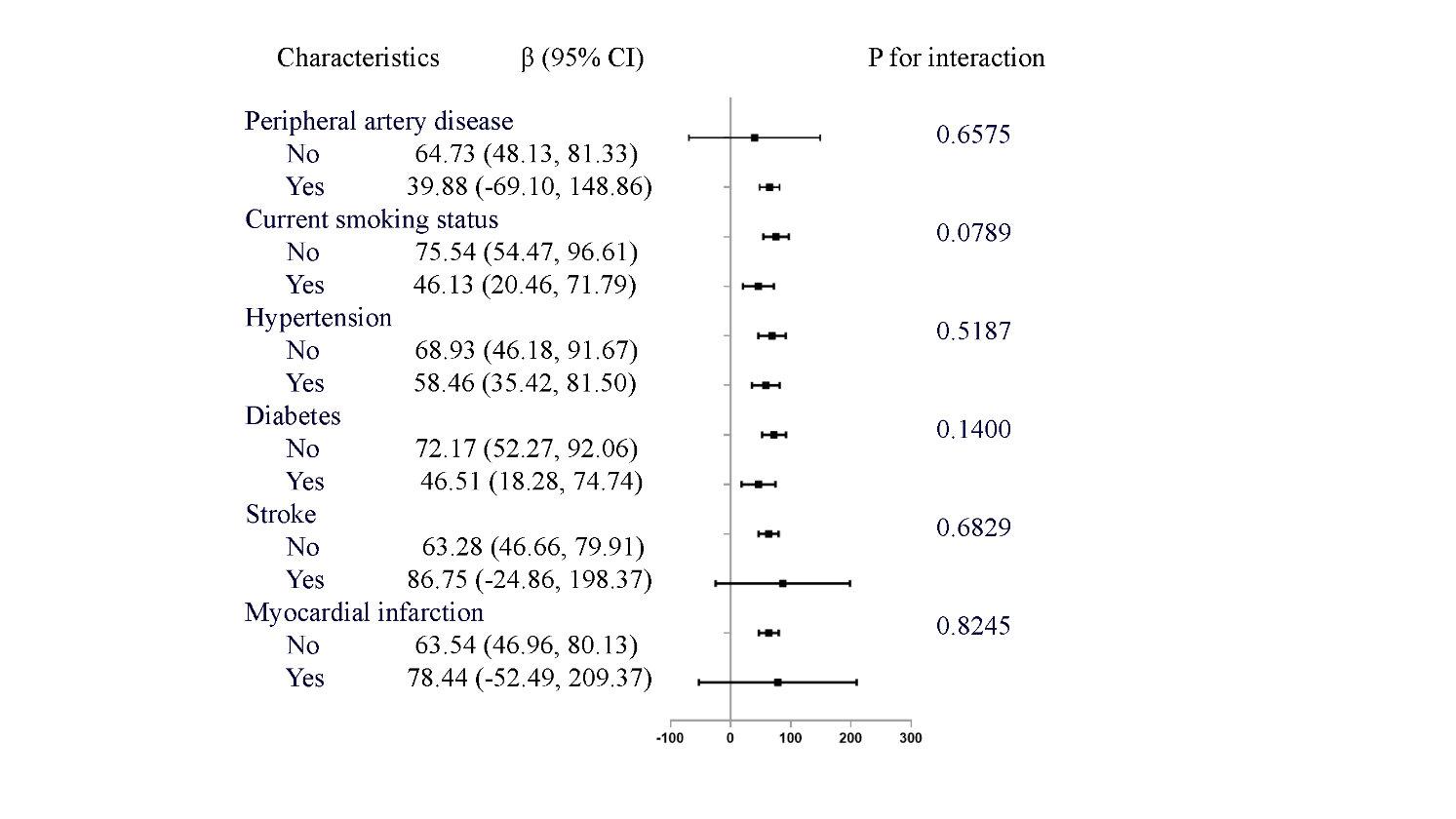


Adjusted, if not stratified, for sex, age, BMI, eGFR, current smoking, current drinking, hypertension, diabetes, using of anti-hypertensive, anti-diabetes, myocardial infarction and stroke.

Supplementary Figure 2. Flowchart for the study.

Community dwellers included

N=8028

taking lipid-lowering drugs

N=1090

missing lipid profiles

N=37

missing baPWV data

N=385

Community residents aged ≥40 years

N=9540
